# Quality-of-life Assessment of Family Planning Adopters through User Perspectives in the District of Karimnagar

**DOI:** 10.4103/0970-0218.42374

**Published:** 2009-01

**Authors:** Kameswararao Avasarala

**Affiliations:** Department of Community Medicine, Prathima Institute of Medical Sciences, Karimnagar, Andhra Pradesh, India

**Keywords:** Family planning adopters, non-FP adopters, Karimnagar district, program indicators, user perspectives

## Abstract

**Background:**

Small families adopting family planning are usually considered happy families. They are expected to lead a better qualitative life. Quality-of-life (QOL) is routinely assessed for knowing patients’ health status. Recently, the QOL concept has become increasingly popular for evaluating the impact of public health interventions. Hitherto, QOL is usually assessed by means of program achievements or indicators, which may sometimes be misleading. Hence, the new culture of QOL assessment by means of user perspectives is now becoming popular. Research Questions: 1) Is the quality-of-life of family planning (FP) adopters better than that of non-FP adopters? 2) Are the user perspectives helpful in QOL assessment?

**Materials and Methods:**

A cross-sectional descriptive study was carried out among 50 FP adopting families and 50 non-FP adopting families from the village of Vutoor and the city of Karimnagar in Andhra Pradesh.

**Sampling Methods:**

Random sampling, Proportions and Chi square test.

**Results:**

Program perspectives revealed a better standard of living for FP adopters because they have amenities like housing, television, and vehicles and less mortality and morbidity (*P* < 0.001). However, they lack positive feelings towards life, general adaptation, personal relationships, and leisure opportunities. Finally, self-assessment by FP adopters themselves revealed no significant increase in their qualitative life after family planning (*P* = 0.05).

**Conclusions:**

While assessing the impact of a health program on quality-of-life, multiple methods of assessments including user perspectives are better than program indicators alone.

## Introduction

Quality-of-life (QOL) assessments are usually carried out for assessing patients’ health status and their quality of life. The QOL concept and its application is now extended to evaluate the impact of public health interventions.(1) While assessing public health interventions, objective indicators and achievements are often used (old culture). These indicators only reveal the provider perspectives and the performance of the program and its programmers. They are one-sided and sometimes may be misleading. On the other hand, user perspectives, the real perceptions and opinions of the beneficiaries will expose the actual components of QOL rather than the objective indices. This evaluation by user perspectives is a new culture of assessment. While the program perspectives provide insight regarding the objective achievements, the user perspectives indicate the qualitative component of achievements by the beneficiaries.(2,3) Unfortunately, the user perspectives are utilized less often.(4) The increased focus of user perspectives on the quality of family planning has led to better understanding of women's reproductive health needs.(5) In this study, the QOL assessment was carried out for the beneficiaries of a national family planning program using user perspectives as well as program achievements. This is being done to assess whether the quality-of-life enjoyed by family planning adopters is better than that of non-adopters.

## Materials and Methods

This comparative cross-sectional descriptive community study was conducted from August to November of 2006 in the district of Karimnagar. The families adopting and not adopting family planning methods were separately listed out from the eligible couples registers of the Urban Health Center in Karimnagar and the Rural Health Center in Vutoor. Fifty families adopting family planning methods and having less than 2 children (small families) and 50 families not adopting family planning and having more than 2 children (large families,) were selected from this list by random sampling. Ten final year medical students of Prathima Institute of Medical Sciences, Karimnagar, conducted door-to-door surveys of these selected families using a pretested questionnaire. The questionnaire included: A) questions on the program indicators, family size, family planning practices, education status, morbidity profile, socioeconomic status, financial debts, and living possessions like vehicle, television, etc. B) Questions on user perspectives relevant to the family as a whole are selected from the following six areas: physical health, psychological health, level of independence, social relationships, environment, and spiritual health. They are positive feelings towards life, general adaptation, self-respect, physical independence, work satisfaction, social support, sexual satisfaction, personal relationships, social integration, physical safety and security, financial capability, life chances, interfamilial relationships, leisure opportunities, and spiritual health. These user perspectives were enquired without asking any leading questions. C) User assessments: as these user perspectives are mostly subjective, the families were asked to assess their QOL by their own perception in terms of percentages of quality they are enjoying and their responses were graded as 0 to 100% (poor QOL 0–25%, below average QOL 25–50%, average QOL 50–75%, and good QOL 75–100%).

QOL is not easy to be assessed as physical, mental, and social well-being have, with varying levels of emphasis and in various combinations, been included in the concept.(6) It is mostly a subjective concept and there is no rule that it can be measured against in a particular way. In this study, it is measured in three ways: firstly, through the program achievements, which will reflect the providers’ perspectives. These may be biased and one-sided. Secondly, it is measured by questioning on 16 individual facets of quality-of-life relevant for families. Here there is a chance for interpretation bias. Finally, it is done by their own self rating of their QOL. Here families were asked to explain their QOL in a single index. Literate families were asked to give their assessment on their QOL in percentages and graded as 25%, 25–50%, 50–75%, and 75–100%. The illiterate families expressed their opinion as quarter, half, three-fourths, and full happiness as QOL measures. In this method, there is no interpretation bias as seen in the second method. It is the family's direct measurement of their QOL in their own words.

A pilot study was carried out and the questionnaire was tested and retested. It was followed by a door-to-door survey using this pretested questionnaire. A total of 100 families were surveyed in 20 days.

## Results

### Differences in the standard of living between two groups

Female literacy among small families is higher than that of large familes (X^2^ _df1_ =23.56, *P* < 0.001 highly significant). Family planning adoption is significantly higher among small families (X^2^_df1_ = 45.12, *P* = <0.001, highly significant). History of mortality in the preceding year is significantly less in small families (X^2^ = 44.08, *P* = <0.001 highly significant). Morbidity in the preceding year, is significantly less in small families (X^2^_df1_ = 29.56, *P* = <0.001, highly significant) [Table 1]. The standard of living is better in small families as indicated by the possession of a good house (X^2^ = 19.03, *P* = <0.001, highly significant), vehicle (X^2^ =33.07, *P* = <0.001, highly significant), television (X^2^_df1_ = 4.29, *P* = <0.05 (significant), and lack of debts (*P* = <0.001) [Table 2].

**Table 1:** Distribution of past mortality and morbidity history in family groups

Family group	Past mortality present (%)	Past mortality absent (%)	Total (%)
Small family group	6 (12)	44 (88)	50 (100)
Large family group	40 (80)	10 (20)	50 (100)
Total	46	54	100 (100)
Family group	Past morbidity present	Past morbidity absent	Total
Small family group	11 (22)	39 (78)	50 (100)
Large family group	35 (70)	15 (30)	50 (100)
Total	46	54	100 (100)

**Table 2:** Distribution of housing characteristics in family groups

Family group	Good house (%)	Bad house (%)	Total (%)
Small family group	38 (76)	12 (24)	50 (100)
Large family group	21 (42)	29 (58)	50 (100)
Total	59	41	100 (100)
**Family group**	**TV available**	**TV not available**	**Total**
Small family group	25 (50)	25 (50)	50 (100)
Large family group	6 (12)	44 (88)	50 (100)
Total	31	69	100 (100)
**Family group**	**Having a vehicle**	**Not having vehicle**	**Total**
Small family group	32 (64)	18 (36)	50 (100)
Large family group	6 (12)	44 (88)	50 (100)
Total	38	62	100 (100)
**Family group**	**Having financial debts**	**Not having financial debts**	**Total**
Small family group	8 (16)	42 (84)	50 (100)
Large family group	34 (68)	16 (32)	50 (100)
Total	42	58	100 (100)

### Differences in the quality-of-life between two groups

Small families are not happy regarding positive feeling towards life, general adaptation, personal relationships, and leisure opportunities [Table 3]. The difference in QOL between small and large families is not significant (X^2^_df3_ = 5.26, *P* = >0.05, not significant) [Table 4]. Almost half of the small families 21 (42%) are feeling bad about their life. Eight (16%) families feel bad because they do not have sons. Three mothers (6%) are feeling that their husbands are neglecting them as they have become obese after undergoing a tubectomy. Two mothers (4%) even complained that their husbands are unfaithful to them after family planning adoption. The remaining eight families (16%) responded that they are not happy and could not explain the reasons.

**Table 3:** Quality-of-life by user perspectives

Facets of life	No. of small families giving a positive response n = 50	No. of small families giving a negative response n = 50	No. of large families giving a positive response n = 50	No. of large families giving a negative response n = 50
Positive feeling	29 (58)	21 (42)	26 (52)	24 (48)*
General adaptation	14 (28)	36 (72)	17 (34)	33 (66)*
Self respect	40 (80)	10 (20)	6 (12)	44 (88)
Physical independence	38 (76)	12 (24)	8 (16)	42 (84)
Work satisfaction	44 (88)	6 (12)	12 (24)	38 (76)
Social support	24 (48)	26 (52)	36 (72)	14 (28)
Sexual satisfaction	42 (84)	8 (16)	6 (12)	44 (88)
Personal relationships	20 (40)	30 (60)	23 (46)	27 (54)*
Social integration	41 (82)	9 (18)	11 (22)	39 (78)
Physical safety and security	39 (78)	11 (22)	12 (24)	38 (76)
Financial capability	43 (86)	7 (14)	12 (24)	38 (76)
Life chances	41 (82)	9 (18)	14 (28)	36 (72)
Interfamilial relationships	36 (72)	14 (28)	12 (24)	38 (76)
Leisure opportunities	23 (46)	27 (54)	21 (42)	29 (58)*
Spiritual health	18 (36)	32 (64)	34 (68)	16 (32)

* Non significant, Figures in parentheses are in percentages

**Table 4:** Self-rating of quality-of-life by the families

Size of the families	Poor QOL (0-25% quality reported)	Below average QOL (25–50% quality reported)	Average QOL (50–75% quality reported)	Good QOL (75–100% quality reported)
Small families	15 (30)	12 (24)	11 (22)	12 (24) = 50
Large families	24 (48)	8 (16)	12 (24)	6 (12) = 50
Total	39	20	23	18 = 100

QOL= Quality-of-life, Figures in parenthesis are in percentages

## Discussion

In this study, family planning adopters are only enjoying a better standard of living but not a better qualitative life than non-adopters. While the program achievements (objective indices) like improved standard of living, less morbidity, and less mortality of small families are indicating that small families are undoubtedly a wealthy and healthy family, user perspectives are revealing that their quality-of-life is not improved. Similar improvement, only in objective indices rather than subjective indices is also seen in the study by Barry and Crosby.(7) The better standard of living observed in small families may be the result of the dual combination of high female literacy and better family planning adoption. The standard of living achieved by the small families in this study is entirely different from qualitative life. The standard of living is giving them just the physical well being but not the actual quality in life as revealed by some of the direct user perceptions of their life.

The strengths of the study: it served its purpose of clarifying that program achievements (objective indices) alone cannot be relied upon while deriving QOL. It also revealed the usefulness of the user perspectives while assessing QOL. They have indeed served as a cross check and revealed the true state of affairs regarding quality-of-life enjoyed by small families. QOL is triple-checked by program achievements (objective indices), user perspectives by third-person interrogation, and user perspectives of the beneficiaries who have their own final say.

The major inherent weaknesses of the study: the instrument of the user perspectives on which the study is entirely based is liable for subjective bias as user perspectives are predominantly subjective. It is subjective to its core. QOL is the subjective evaluation of life as a whole.(8) It is an all-encompassing and subjective concept.(9) It is a multidimensional construct encompassing perceptions of both positive and negative aspects of dimensions such as physical, emotional, social, and cognitive functions, as well as the negative aspects of somatic discomfort and other symptoms produced by a disease or its treatment.(10,11) It is the extent to which hopes and ambitions are matched by experience.(12)

It is the individuals’ perceptions of their position in life taken in the context of the culture and value systems where they live and in relation to their goals, their expectations, standards, and concerns.(13) it is the appraisal of one's current state against some ideal.(14) it is things people regard as important in their lives.(15)

Hence, it is not an easy task to measure QOL as it is multidimensional, dynamic, and individualistic. The number of concepts, dimensions, and components we are measuring or can measure is very difficult to ascertain. The only resort is to measure as many components as possible as we cannot measure the whole quality-of-life. It is modestly tried in this study. The determinants of QOL are specific to individuals; the importance attached to those determinants will be influenced by an individual's expectations and aspirations as well as by their own belief system, their cultural belief system, and socio-demographic factors such as age, sex, socioeconomic status, education, geographical location, and marital status. A true assessment of QOL can only be achieved using weights for individuals.(16) QOL is dynamic, (not static) changing over time and over a person's life. It arises from a person's interaction with their environment. It is experienced differently from person to person, but has the same components for everyone.(17) This individual nature of QOL and its dynamic nature makes the individual weights less reliable when its comes to the QOL assessment of the whole family, as different family members may rate QOL differently. Lastly, there is a chance that some of the facets of life used to assess QOL in the study may get intermixed as the opinions of the users are purely subjective and depend on their understanding of that facet of life.

Practical applications of the study: subjective QOL measures are now increasingly used to supplement objective clinical or biological measures of disease to assess the quality of service, the need for health care, the effectiveness of interventions, and cost utility analyses.(18) The subjective information is necessary to complete the QOL picture and to enhance the interpretation of objective data(19) as satisfaction of the users is also closely related to quality of services.(20) User perspectives have to be widely used for quality assessment in all situations, not only in chronically ill patients. User opinions and experiences have to be considered while defining priorities for taking health action.(21) They can be routinely included in evaluation of any health program in addition to the program perspectives. They have to be included as part of an in-built evaluation for all the national health programs. User perspectives have an added advantage of increasing community participation and working together with the providers, which is essential for making any program successful.(22)

Future research in QOL assessment must incorporate the perspective of the individual to enable valid conclusions to be derived based on content that is relevant to the individual being assessed, thus informing management decisions, policy, and practice more meaningfully.(23) But further research is essential to refine all the presently available subjective instruments of QOL assessment and make them sharper and specific for quality-of-life assessment to be applied to all situations.

## Conclusions

There is no doubt in concluding that the user perspectives can strengthen and improve the quality of any assessment. The subjective information is necessary to complete the QOL picture and to enhance the interpretation of objective data.(23) The user perspectives, which are the direct perceptions and opinions of the beneficiaries, may describe the true state of affairs of QOL impact on beneficiaries in a more useful way for future program planning.
